# The impact of Bis-GMA free and Bis-GMA containing resin composite as posterior restoration on marginal integrity: a randomized controlled clinical trial

**DOI:** 10.1186/s12903-023-03759-5

**Published:** 2023-12-19

**Authors:** Sara Ahmed Reda, Yasser Fathi Hussein, Mona Riad

**Affiliations:** 1 Operative Dentistry Department, Faculty of Dentistry, Minia University, Minia, Egypt; 2 Dental Biomaterials Department, Faculty of Dentistry, Minia University, Minia, 61519 Egypt; 3 Conservative Dentistry Department, Faculty of Dentistry, Cairo University, 11 El-Saraya St, Manial, Cairo, 11553 Egypt

**Keywords:** Composite resin restoration, BIS-GMA containing composite, BIS-GMA free composite, Marginal integrity

## Abstract

**Background:**

There have been concerns surrounding the utilization of Bis-GMA, a type of bisphenol A (BPA) derivative, within the dental industry. The aim of this study was to compare the performance of bulk fill Bis-GMA-free resin composite class II restorations in respect of its marginal integrity in comparison to bulk fill Bis-GMA-containing resin composite class II restorations over a 12-month period in a parallel clinical trial utilizing a split-mouth, double-blind, randomized strategy.

**Methods:**

20 patients participated in this study. Each patient has received one pair of class II posterior restorations, Bis-GMA-free (Admira fusion x-tra), and Bis-GMA containing (x-tra fil) on each side of the mouth (split-mouth strategy), (n = 40). The restorations’ marginal integrity was evaluated based on Ryge’s criteria (modified USPHS) at baseline (after 1 week), as well as 1 month, 3 months, 6 months, 9 months, and after 12 months of follow-up by two calibrated examiners. The statistical analyses utilizing the Friedman and Wilcoxon tests, the significance level was adjusted to 0.05.

**Results:**

Following the 12-month period, all patients attended the recall visits to evaluate the restorations. The Wilcoxon signed-rank and Friedman tests, revealed that both types of bulk fill had 100% of Alpha (A) scores at baseline and after 1 month with no significant statistical differences. After 3, 6, 9, and 12 months, both tested bulk fill restorations showed Bravo (B) score with Bis-GMA free 10% and 5% for Bis-GMA containing with no statistically significant difference (*p* ≤ 0.05) for clinical marginal integrity parameter in USPHS criteria.

**Conclusions:**

Bis-GMA-free resin composites demonstrated satisfactory, marginal integrity compared with Bis-GMA-containing resin composites within 12 months.

**Trial registration:**

The protocol of the current study was registered at www.clinicaltrials.gov, with the identification number NCT05480852 on 29/07/2022. All procedures involving human participants were performed in accordance with the ethical standards of the Research Ethics Committee of the Faculty of Dentistry, Minia University, Egypt, under the approval number 419 on 27/06/2020.

## Background

In recent decades, resin composites (RCs) have demonstrated significant potential for replacing amalgam in dental restorations. In addition to their aesthetic appearance, composite restorations necessitate no significant preparations, preserve tooth structure, and have demonstrated promising clinical performance within posterior teeth [1, 2].

Several factors contribute to the success of direct composite restoration, such as the material properties, cavity preparation design, and the technique utilized for material application [3]. Various techniques for placing composite resin were suggested to enhance the restorations clinical outcomes and mitigate the polymerization shrinkage adverse effects and the resulting stress [4]. To eliminate the impact of shrinkage stress and minimize the occurrence of gap formation, several strategies have been employed. These include procedures like resin restorations indirect placement, utilization of a flowable resin liner, careful control of curing light intensity, and implementation of incremental layering methods [5].

The techniques for composite application involve layering and using thin 2 mm polymerization increments are widely used [6–8] but it’s a time-consuming activity. Layering technique carries the inherent risk of introducing contaminants or air bubbles between the increments specially in extensive cavities in posterior teeth, [9]. To streamline the process of inserting composite material to the cavity and facilitating the polymerization, manufacturers provide bulk-fill type composite resins. The ability to apply a larger amount of composite material in a single layer, with depths reaching 4 mm, contributes to the streamlining and time-saving advantages of bulk-fill type restorations placement. This enables faster work and reduces the clinical steps involved [7]. Nonetheless, optimum bulk-fill composite would possess the potential for being applied to a high C-factor preparation while displaying negligeable polymerization shrinkage stress, all the while maintaining a significant level of curing [10].

Recently, significant efforts have been dedicated to enhancing filler technology, leading to advancements in the composite materials’ esthetic and mechanical properties. As a result, we now have nanohybrid and nanoparticle-containing composites that offer improved characteristics [11]. However, few changes were performed in relation to the organic matrix, and many traditional di-methacrylate monomers are still in use. Since its fabrication by Bowen in 1956, BIS-GMA (bisphenol A-glycidyl methacrylate) has denoted the primary monomer utilized in composite compositions [12]. It typically comprises three components: a photoinitiator system, an organic resin matrix, and inorganic fillers treated with the bonding agent. BIS-GMA monomer is highly viscose, therefore, monomers with a lowered molecular weight were added to the mixture to accomplish the proper viscosity for the ultimate clinical preparation [13]. These diluent monomers lead to an elevation in composite water sorption and polymerization shrinkage [14]. Furthermore, the eluted unreacted monomer from the cured material renders it more cytotoxic toward pulp cells [15]. Consequently, there has been an exploration of new monomers targeting to enhance the composite restorative materials characteristics.

Organically modified ceramics are fabricated using polycondensation and hydrolysis processes (sol-gel processing) to develop molecules with organic lateral chains and lengthy inorganic silica chain backbone [16]. Regarding Bis-GMA, the molecule contains additional methacrylate groups capable of forming bonds [17, 18]. As a result of creating an intensely crosslinked polymer network, it is anticipated that the composites containing ormocer will exhibit a higher degree of conversion, as well as enhanced wear resistance and tensile strength [19].

Nevertheless, the initial iteration of ormocer composites featured ormocer molecules and conventional lowered molecular weight di-methacrylate monomers, serving as diluents. The inclusion of these diluents can hinder the anticipated outcomes, and there were no evident benefits occurred when utilizing 1st generation ormocer-based fillings compared to conventional composites [20, 21]. A pure ormocer composite has been recently established. ORMOCER restorative material, *Admira fusion x-tra* with (84% by wight filler nano-hybrid glass ceramics, silica particles. As per the manufacturer’s specifications, the composition of the ormocer composite does not include any diluent methacrylate monomers. Instead, they have developed unique ormocer molecules with varying viscosities. In contrast to methacrylate composites, this novel ormocer material exhibits potential benefits such as reduced water sorption and polymerization shrinkage [22, 23]. Additionally, this novel material demonstrates increased microhardness and increased degree of conversion [24].

In vitro studies employ various techniques to evaluate the occurrence of microleakage between the filling material and the tooth tissues [10]. While achieving a perfect marginal seal may be challenging in clinical practice, clinicians should strive for a high-quality marginal adaptation as their primary objective. Conducting a clinical assessment of the newly introduced bulk-filling restorations is crucial to evaluate factors such as marginal adaptation, anatomical shape, and the potential for margin discoloration. Additionally, monitoring annual failure rates provides valuable insights. Marginal integrity stands out as one of the most crucial parameters that determine the quality of materials used for restoring lost tooth tissues.

The evaluation of dental restorations is based on a variety of clinical standards. The most popular set of standards is known as Ryge’s criteria [25], often referred to as the United States Public Health Service (USPHS) standards. Nevertheless, there are insufficient documented clinical trials comparing the performance of bulk-fill Bis-GMA-free composites to bulk-fill Bis-GMA-containing composites. In the present time, a multitude of promising novel materials has become affordable, contributing to a highly bewildering selection process. The study aims to appraise marginal integrity of class II cavities composite restorations formulated from 2 bulk-fill materials (a nano-hybrid ORMOCER resin composite material Bis-GMA free, ***Admira fusion x-tra*** to conventional methacrylates-based one Bis- GMA containing, ***x-tra fil***). This clinical study evaluated in 40 class II posterior teeth restorations using modified Ryge’s criteria for marginal integrity (USPHS) [26]. The tested null hypothesis indicates that both bulk-fill composite resins reveal similar marginal adaptation.

## Methods

### Study design and settings

**Fig. 1:**
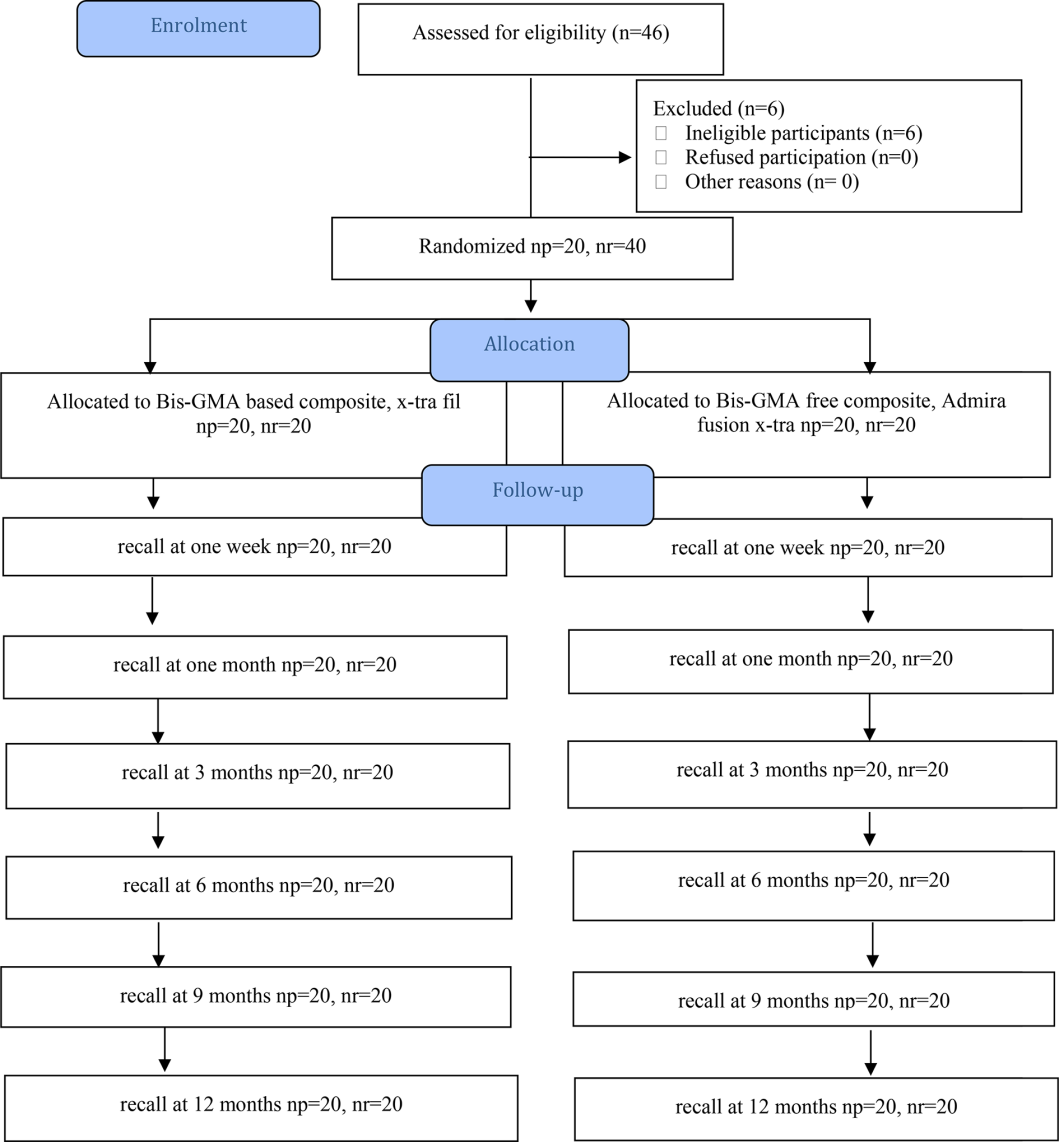
Flowchart of the current investigation (Consolidated Standards of Reporting Trials [CONSORT] 2010)

The current study was a split-mouth and double-blind longitudinal, prospective randomized clinical trial (RCT) with a follow-up of 12 months. Both the clinical examiner and the participant were blinded to the intervention. The Research Ethics Committee approved the research proposal (Ref. no. 419 on 27/6/2020). Furthermore, the research proposal has been registered in the *Clinical Trials Registry* with the identification number NCT05480852 on 29/07/2022. The study’s reporting was adhered to the Consolidated Standards of Reporting Trials (CONSORT) statment [27] Fig. 1. Written consent was received from all participants. This research was carried out by a single operator at the outpatient clinic of the Department of Operative Dentistry, Faculty of Dentistry, Minya University, between May 2021 and June 2022.

The PICO query was posed, and the following constraints have been established: P: adult patients with two class II cavities; I: Bis-GMA free (pure ormocer) composite restoration; C: Bis-GMA containing (methacrylate) composite restoration; and O: evaluation of marginal integrity parameter according to USPSH criteria. The primary research inquiry addressed in this study was to determine if there is a similar level of marginal integrity between class II restorations created using pure ormocer composite and those made with methacrylate composite, as evaluated according to USPSH criteria?

### Sample size calculation

The sample size was determined utilizing prior research’s composite restoration clinical success rate (93% at 12 months) [28]. Eighteen restorations were used in previous research that examined posterior tooth restorations with a 0.05 significance level, power of 80%, and equivalency limit of 20%. With the possibility of dropouts, 20 restorations from each group have been accomplished (for a total of 40 restorations), and thus, 20 patients have been accepted, given the split-mouth strategy utilized.

### Eligibility criteria

Patients between 18 and 40 years of age had carious lesions in both proximal and occlusal surfaces that were detected clinically and evaluated by X-ray. The antagonist and opposing teeth make contact, have vital pulp, no aching symptoms, have a proven history of no hypersensitivity in the teeth requiring restoration, and have good oral health.

Moreover, patients with heavy bruxism habits, engaged in clenching, showed evidence of wear facets on teeth, took analgesics that could alter their normal pain perception level, suffered occlusal disturbances, experienced temporomandibular joint problems, or underwent orthodontic treatment were excluded.

### Randomization: sequence generation and allocation concealment mechanism

A randomization list has been prepared using a web program (www.randamization.com). Each patient was assigned an identifying code (P1; P2,…. P20), and the two composite options (F -Bis-GMA free RC and C -Bis-GMA containing RC) were chosen randomly from a list. Each patient has received one pair of class II posterior restorations, one Bis-GMA-free, Admira fusion x-tra and one with Bis-GMA containing, x-tra-fill RC restorative on each side of the mouth (split-mouth strategy). A blocked list was formed, and a randomization code was developed relying upon two restoration options. Similarly sized and positioned cavities were also chosen for each pair. A secretary who was not engaged during the clinical procedures prepared separate sealed opaque envelopes for each patient and included a link between the randomization code and the type of restoration used. Consequently, the operator initiated the restoration process by selecting one of the two opaque sealed envelopes containing the randomization code for the first quadrant to be restored. The chart of the patient utilized only for the recalling included the codes of randomization, broken shortly after the clinical assessment was completed.

### Clinical procedure

Periapical radiographs of teeth requiring treatment were obtained prior to the initiation of restorative procedures. A Parkell Pulp vitality tester (Parkell Electronics DN, Farmingdale, NY, USA) has been used to record the teeth vitality test results. All restorations were acquired by one operator, and the primary investigator supervised all clinical processes. Depending on the accessibility, a Rubber dam was placed either prior to the surgical procedure or after the cavity was opened following anesthesia. The carious tissue and all undermined enamel were removed to allow access to the cavity using a high-speed carbide bur #245 (1.6 mm length and 0.8 mm diameter) with sufficient air/water spray (Komet dental Gebr brasseler GmbH and Co lemgo, Germany). Using a selective caries removal, deep caries were removed using a hand excavator and low-speed tungsten carbide burs. The excavation of the preparation floor was primarily guided by probing with an explorer and observing the dentin’s color. The adhesive preparation design adhered to minimally invasive dentistry principles. Each patient received two restorations under similar clinical conditions.

Futurabond M (Voco), a universal dual-cure self-etching adhesive, was administered to all preparations following the manufacturer’s guidelines. The adhesive was applied actively for 20 s, followed by a soft air blow for 5 s and light curing for 10 s. The restoration procedures involved the utilization of a separation ring, a pre-contoured metallic sectional matrix, and a wooden wedge (Unimatrix System, TDV, Pomerode, SC, Brazil). A periodontal probe was utilized to assess the cavity depth. A universal shade was applied in a single bulk increment when the preparation was up to four millimeters deep. The composite was cured with light for the 20s after shaping. Carbon paper was used to make a post-occlusal adjustment, while dental floss and interproximal radiographs were utilized to assess the quality of the cervical adaptation and interproximal contacts. The restorations were finished with fine-grain diamond burs (KG Sorensen, S˜ao Paulo, SP, Brazil) under water cooling. Abrasive strips (3 M ESPE / St. Paul, MN) were utilized to remove any excess at the proximal surface. Fine and super-fine diamond points with abrasive rubber points (Dimanto, Voco) were made at the proximal surfaces with fine-grained strips during the same appointment immediately following the restoration procedures.

### **Calibration procedures for clinical assessment**

While the operator was aware of the intervention, examiners and patients were unaware of the material assignment. The quality of the margin of composite restoration was clinically assessed by two calibrated, experienced, blinded examiners following Ryge’s criteria (USPHS) [26]. At baseline and during the recall appointments, intraoral photographs were captured using a Canon D2000 camera with a Macro lens (Canon, Tokyo, Japan). Restorations evaluations were accomplished after a week and 1,3, 6, 9, and 12 months following the USPSH criteria. The restorations were visually examined under the dental unit’s overhead light with magnification aids. Cotton rolls were placed to isolate the tooth and maintain a dry field for examination. Specialized probes were used to analyze the marginal adaptation. Two special probes (Deppeler, Rolle, Switzerland), one with a 150 μm tip and the other with a 250 μm tip, were employed to classify the marginal gap width. Restorations were assigned a score of either “Alpha” to denote ideal clinical scenario, “Bravo” for clinically acceptable, “Charlie” for clinically unacceptable necessitating replacement, or “Delta,” denoting mobile, fractured, or missing restorations urgently requiring replacement. Furthermore, follow-up appointments incorporated bitewing radiographs to supplement clinical assessment. The marginal integrity of the restorations was checked after a week (baseline), 1, 3, 6, 9, and 12 months. Any inconsistencies in evaluation results were resolved through consensus between both evaluators.

### Statistical analysis

The clinical evaluation scores were compared utilizing the Wilcoxon signed-rank test to estimate significant differences among the two materials at an inspection time or within a provided material through time. The variations over time within each group were evaluated using Friedman’s test. The normality of the numerical data was assessed by examining the distribution of data and utilizing normality tests (Shapiro-Wilk and Kolmogorov-Smirnov).

## Results

The restorative procedures were implemented exactly as planned, and no modification was performed. 6 of 46 subjects were not enrolled in the study because they did not fulfill the inclusion criteria. The present study was conducted on 20 patients: 12 females (60%) and eight males (40%). There was no statistically significant difference between teeth. Table 1 exhibits the data regarding the characteristics of patients and restored cavities. The attendance rate for recall evaluations was 100% after 7 days, 1 month, 3 months, 6 months, and 12 months. The quality of the composite restoration margin was classified based on the USPHS criteria as **Alpha (A)**: acceptable; margin did not dissemble; probe failed to catch. **Bravo (B)**: standard; probe catches on the margin but does not encounter any gap, liner, or dentin. **Charlie (C)**: it indicates that, upon probing, the probe catches on the margin and gap, thereby exposing the liner or dentin. **(D)**: unsatisfactory; restoration is broken or missing. Table 2 showed that Bis-GMA-free RC and Bis-GMA-containing RC both had 100% of Alpha (A) scores at baseline and after 1 month with no significant statistical differences. After 3, 6, 9, and 12 months, showed a reduction in (A) score for both types of restorations and showed Bravo (B) score with Bis-GMA free 10% and 5% for Bis-GMA containing with no statistically significant difference. At the 3-month mark, only two restorations exhibited minor fractures, one made with pure Bis-GMA free composite and the other made with Bis-GMA containing composite. However, these fractures were insignificant and did not necessitate restoration replacement.

**Table 1:** Frequencies (n), percentages and results of Wilcoxon signed-rank test for comparisons of demographic data in the two groups

	BIS-GMA-free composite (n = 20)		BIS-GMA-containing composite (n = 20)		*P-value*
	n	%	n	%	
Tooth					
Mandibular premolars	1	5	1	5	0.750
Mandibular molars	10	50	9	45	
Maxillary premolars	3	15	3	15	
Maxillary molars	6	30	7	35	

*Significant at P ≤ 0.05

**Table 2:** Descriptive statistics and results of Wilcoxon signed-rank test for comparison between marginal adaptation scores in the two groups and results of Friedman’s test for comparison between marginal adaptation scores at different follow-up periods within each group

Time	BIS-GMA-free composite (n = 20)		BIS-GMA-containing composite (n = 20)		*P*-value
	n	%	n	%	
Baseline					
Alpha	20	100	20	100	**1**
Bravo	0	0	0	0	
1 month					
Alpha	20	100	19	95	**0.317**
Bravo	0	0	1	5	
3 months					
Alpha	18	90	19	95	**0.564**
Bravo	2	10	1	5	
Charlie	0	0	0	0	
6 months					
Alpha	18	90	19	95	**0.564**
Bravo	2	10	1	5	
Charlie	0	0	0	0	
9 months					
Alpha	18	90	19	95	**0.564**
Bravo	2	10	1	5	
Charlie	0	0	0	0	
12 months					
Alpha	18	90	19	95	**0.564**
Bravo	2	10	1	5	
Charlie	0	0	0	0	
*P-value*	**0.075**		**0.416**		

*significant at P ≤ 0.05

## Discussion

While RC incorporating Bis-GMA provide benefits such as improved aesthetics, ease of handling, and mechanical sturdiness that have made them essential in restorative dentistry, opportunities for further optimization still exist with these materials. In healthy tooth structures, polymerization shrinkage and resulting contraction forces can cause post-operative sensitivity, secondary caries, marginal discoloration, displacement of cusps, and even cracks. Consequently, removing or minimizing the volumetric contraction amount during polymerization [29] caused by the monomer phase is among the most significant problems in dental composite fabrication. Therefore, novel monomers have been investigated to develop composite restorative materials with improved characteristics.

In more recent years, a new class of restoration materials called ormocers has emerged based on Ormocer chemistry. Notably, these ormocer-based restoratives were purportedly devoid of bisphenol A or any methacrylate-derived monomers [30]. The goal in developing ormocer composites was to decrease polymerization shrinkage through modifications to the organic matrix composition and augmenting the silicate filler load.

According to the manufacturer, the new bulk-fill ormocer does not contain any congenital monomers beyond those present in the ormocer matrix composition. The material utilizes a nanohybrid filler technology, containing 84% inorganic filler by weight. Through a combination of silicon dioxide as the inorganic base along with polymerizable organic components, it allegedly merges the resilience of glass with resin-like handling characteristics. This material aims to enhance the aesthetics and abrasion resistance, reducing polymerization shrinkage, surface roughness, and caries protection. Additionally, as it does not incorporate Bis-GMA or typical methacrylates, concerns around cytotoxicity are absent since the material is purportedly inert, thereby potentially improving biocompatibility [31].

Because the bulk-fill RCs contained polymerization modulators that reduced tension and contraction at the bonded interface, a methacrylate-based bulk-fill composites were chosen over incremental composites for testing [32–34] and comparing with ormocer-based one. Inserting thicker increments can decrease air space integration, providing a highly uniform restorative unit [32]. Furthermore, enhanced translucence and additional reactive photoinitiators [32] enable a deeper cure. The elevated reactivity of this material allows inserting thick increments (4–5 mm), with consistent polymerization and conversion degree. These elements are necessary to maintain good mechanical qualities, prolonging the restorations’ lifespan [35, 36].

In addition to the lowered polymerization shrinkage, the decreased maximum force rate (R-max) determined for the Bis GMA free (ormocer-based) bulk-fill RC likely contributed significantly to its demonstrated favourable shrinkage force behaviour. The lowered *R-*max value specifies that Bulk Ormocer produces polymerization-stimulated forces at lower rates, allowing more time for the emerging polymer network to re-arrange spontaneously throughout the early curing stage. This provides a window for the partial dissipation of developing shrinkage forces through polymer chain relaxation and viscous flow before restraining mobility via vitrification [37–39].

By limiting the age range, researchers can reduce the variability in their data and increase the reliability of their results. This is because age can influence many factors related to dental health, such as the condition of the teeth and gums, the presence of diseases, and the response to treatment. The age group of 18–40 is a critical period where individuals often have permanent teeth but have not yet experienced significant age-related dental issues. Therefore, studies targeting this age group can yield results that are relevant to a large population. Younger and older individuals may have different risk profiles, and including them in studies may raise ethical issues. For example, children and adolescents are still growing, and their teeth and jaws are still developing, so the effects of treatments may be different for them. Older adults may have more health complications that could complicate the study or put them at risk. It might be easier to recruit participants in the 18–40 age group, and they may be more likely to complete the study.

The present investigation assessed the marginal adaptation of the restorations with resin composite depending upon ormocer matrix technology *(Admira Fusion x-tra)* and Bis-GMA-containing composite *(x-tra fil).* This study found no significant statistical differences within the scores of marginal adaptations in the two groups over time. Restorations performed with ormocer- based RCs would exhibit the same marginal adaptation as restorations performed with conventional methacrelate RCs. The results were supported by da Veiga et al. 2016 [40], indicating no considerable discrepancies regarding the distribution of scores for the marginal integrity between follow-up periods in the intervention and control groups (p = 0.074). Excessive polymerization contraction strains at the tooth-restoration contact might lead to marginal defects. Slow hydrolysis, which leads to deterioration of the resin/bond interface, may potentially contribute to the statistically significant rise in marginal deficits over time [33].

Hayashi et al. [41] stated that additional standardization of methods would be necessary for clinical studies to achieve actual comparability of their findings. Moreover, they demonstrated that clinical studies are ineffective in the development of “evidence-reliant” dental medicine. Recent advancements within the field of composite technology have demonstrated limited success in clinical investigations. Previously, certain materials marketed as having easy handling or “amalgam-like” clinical procedure have been proven inadequate when subjected to clinical testing [42]. P. Bottenberg et al., [43] evaluated the performance of hybrid ormocer based restorative systems and conventional bis-GMA containing composite restorative system in occlusal stress-bearing restorations over 3 years and found that within the cohort of class II restorations, there was no clinically substantial disparity in failure incidents over the 3-year timeframe between restorations fabricated using the ormocer-based versus bis-GMA-containing materials. In a 3-year clinical assessment, SH Mahmoud et al. [44] assessed and compared the performance of four restorative materials: an ormocer composite (Admira), a nanoceramic composite (Ceram X), a nanofilled resin (Filtek Supreme XT), and a microhybrid composite (Tetric Ceram), when employed in restoring Class I and II cavities. The study found only minor changes to all materials over the three-year period, with no detectable differences between their initial and three-year performance levels. Failure occurred in two ormocer, one microhybrid, and one nanofilled molar restorations specifically due to loss of retention. Statistically, no significant variations (p > 0.05) occurred in clinical performance of the tested materials. The results met the American Dental Association (ADA) acceptance criteria, which specifies < 5% failure rate at two years for restorations undergoing clinical inspection [45].

Efes and others [46] investigated the clinical efficacy of packable ormocer, nanofilled, and hybrid composites for treating occlusal cavities created using a less extensively invasive approach. Despite the cavity’s large configuration factor, it was reported that both materials exhibited satisfactory clinical performance.

In a recent clinical trial lasting for two-year, class I ormocer, nanohybrid, and nanofilled RCs were compared to microhybrid composites. All these restorative materials performed well in clinical trials [47]. The Bis-GMA-free experimental bulk-fill resin composite, which is based on ormocer, exhibited minimal shrinkage force and linear polymerization shrinkage, which is likely attributable to the presence of inorganic-organic copolymers (e.g., TEGDMA, UDMA, and Bis-GMA) in the resin system, and to the lower quantities of organic resin in comparison to di methacrylate-based composites [48].

According to a study [49], the hybrid resin composite system demonstrated superior marginal integrity compared to ormocer in both unloaded and loaded restorations, specifically along the occlusal and cervical margins. In line with these findings, our study revealed that the nanoceramic and nanofilled composites achieved a clinically ideal marginal adaptation rate (Alpha) of 100%, while the microhybrid composite attained a rate of 97.5%. On the other hand, the ormocer exhibited a consistent rate of 97.5% at both one and two years. Nevertheless, after three years, all materials experienced a change in this criterion, but without any clinically significant differences observed.

Tetric Ceram, as well as its precursor Tetric resin composite, have been assessed in long-term clinical studies [30, 50], which revealed favorable clinical performance in posterior teeth. In a study by Rosin and others [30], the clinical efficacy of ormocer restorations was examined, specifically focusing on marginal integrity and marginal discoloration. The results revealed excellent outcomes in these aspects after a six-month period. According to Bottenberg and others [43], the ormocer-based composites (Admira and Definite) exhibited comparable performance to the traditional microhybrid bisphenol A diglycidyl ether dimethacrylate-based composite within occlusal stress-bearing cavities, except for the poor color matching.

Florian Beck et al., [51] assessed the efficacy of two direct composite resins within stress-bearing Class I/II cavities with cuspal-coverage. The restorations were accomplished by students in molar and premolar teeth of 456 patients. The majority of failures observed in the study were attributed to issues related to the marginal adaptation and integrity of the fillings. Furthermore, the occurrence of failures was significantly influenced by factors such as the number of teeth receiving treatment by each patient, patient age, restoration mesio-distal extension, and the position of the tooth. There was no considerable consequence on the failure rate observed in relation to sex, material type, filling’s bucco-lingual extension, earlier root canal treatment, or cuspal-coverage. However, it was found that patients who attended the initial recall visit were notably older and had a higher number of fillings compared to those who did not attend. No considerable disparity in failure rates was observed between bis-GMA-based and ormocer-based restorative systems within the one-year timeframe. However, to acquire definitive clues regarding the long-term efficacy of the composite resin systems, a more extended observation period is warranted.

The Ryge Criteria or USPHS Criteria is a set of clinical constraints devised by Gunnar Ryge [52] to judge the quality of composite restorations. The California Dental Association adapted these criteria for quality assessment and called them “Modified USPHS Criteria or USPHS/CAD Criteria” [53]. In contrast, the purpose of this grading system was to emphasize acceptability (yes/no) rather than achievement level. In the current investigation, the marginal adaptability of the evaluated restorative systems has not changed after one year compared to a baseline.

Göstemeyer et al. [54] indicated the limited sensitivity of the USPHS in entirely reflecting the restorations clinical success. Clinical trials incorporating supplementary criteria alongside the USPHS system often reveal substantially higher failure rates, surpassing those obtained solely through the utilization of the USPHS criteria by more than fourfold. An alternative to the USPHS system is the FDI criteria, which offers the possibility of simplification by combining scores 1 to 3 into a single category representing clinically good/satisfactory/acceptable. the evaluation of restorations involved separate utilization of the FDI and USPHS criteria. The FDI criteria were chosen to align with the emerging trend of their application in restoration assessment, while the USPHS criteria enabled comparisons with prior studies. Notably, significant disparities were observed when contrasting respective categories between the FDI and USPHS criteria. These discrepancies encompassed parameters such as roughness (USPHS) versus surface gloss/luster and roughness (FDI), as well as marginal adaptation. In the two categories, the “acceptable” score percentage was substantially elevated for the USPHS criteria. Both systems demonstrated comparability in the remaining categories. These discrepancies could potentially be explained by variations in the evaluation score parameters. The assessment of marginal adaptation revealed contrasting criteria between the FDI and USPHS systems. The FDI criteria regarded small gaps (< 150 μm) and marginal fractures that could be resolved through polishing as successful (score 2). In contrast, the USPHS criteria considered any detection of explorer catch as acceptable (bravo), irrespective of visible evidence of penetrable gaps.

In this study, the ormocer materials were discovered to be on par with a methacrylate composite, although they did not exhibit superiority. This could be attributed to the recognition that success or failure is not solely determined by material properties. Leloup et al. [55] indicated that the adhesive force among composite and dentin is influenced by various factors, including the quality and origin of the tooth’s hard tissues, as well as the dentinal tubules’ direction and diameter. The clinical success of the restorations is determined by criteria like the patient’s caries risk, the extent and placement of the restoration, and the material quality [56]. Other factors, including socioeconomic condition, parafunctional behaviors, and operator experience, directly affect the durability of dental restorations in the oral cavity. Dentists deal with these issues on a daily basis and require scientific data to determine whether or not to use innovative materials and treatments [57].

## Conclusion

Within the limitations of this study, it can be concluded that Bis-GMA free resin composite exhibited comparable performance to Bis-GMA containing. Although the marginal integrity was affected over time however the change deemed clinically acceptable. More clinical trials with follow-up longer than 12 months are needed to evaluate the prolonged efficacy of these RCs.

## Data Availability

The datasets used and/or analysed during the current study are available from the corresponding author on reasonable request.

## Abbreviations

BPA: bisphenol A
Bis-GMA: bisphenol A-glycidyl methacrylate
UDMA: Urethane acrylate methacrylate resin
TEGDMA: Triethylene glycol dimethacrylate
USPHS: United States Public Health Service
